# Promoting Diversity in the Recruitment of a Youth Advisory Council in the Mental Health and Addictions System

**DOI:** 10.1111/hex.70208

**Published:** 2025-03-02

**Authors:** Deewa Anwarzi, Nhi Man (Jennifer) Tran, Thalia Phi, Roula Markoulakis

**Affiliations:** ^1^ Sunnybrook Research Institute Toronto Ontario Canada; ^2^ Department of Psychology Neuroscience and Behaviour McMaster University Hamilton Ontario Canada; ^3^ Family Navigation Project Sunnybrook Health Sciences Centre Toronto Ontario Canada; ^4^ Department of Occupational Science and Occupational Therapy Faculty of Medicine University of Toronto Toronto Ontario Canada

**Keywords:** equity diversity and inclusion, lived experience, navigation, youth advisory council, youth engagement, youth‐led

## Abstract

**Introduction:**

There is increasing interest in youth advisory councils (YACs) within the mental health and addictions (MHA) system; however, little is known about methods to ensure diversity of perspectives on these councils. Diverse perspectives are critical for ensuring that youth MHA organizations are equipped to support youth needs.

**Methods:**

A youth‐led YAC recruitment initiative was developed and implemented at a GTA‐based youth MHA navigation service: the Family Navigation Project.

**Results:**

Specifically, this paper highlights the efforts of three youth staff and volunteers who championed a YAC recruitment plan focused on promoting equity, diversity, inclusion (EDI) and accessibility, youth engagement (YE), and lived experience/expertise (LE).

**Discussion:**

This paper is written from the perspective of the youth recruitment team and provides important insights for MHA services interested in ensuring EDI, accessibility, YE and LE on a new or existing YAC, and researchers studying YE in MHA care.

## Patient or Public Contributions

This initiative was developed and implemented by youth with lived experience. The final manuscript was also co‐written by these youth.

## 1 Introduction

On‐going research suggests considerable inadequacy in youth access to mental health and addiction (MHA) care [1]. An evidence‐based strategy for improving youth access to MHA care is through youth engagement (YE) [2]. YE has been shown to improve youth MHA outcomes. One way that current MHA services aim to promote YE is through youth advisory councils (YACs), as these councils systematically embed youth voices into the structure of organizations [3]. The effectiveness of YACs lies in their ability to amplify diverse perspectives, as it is through the adoption of these diverse perspectives that MHA services can better meet the dynamic and evolving needs of youth. However, a key gap that remains both in research and practice is determining how to best promote diversity across councils.

This paper seeks to bridge this gap by centering the discussion on youth perspectives regarding diversity and its role on the development of YACs. In particular, it highlights the perspectives and efforts of three youth who, through their roles at the Family Navigation Project (FNP)—a free GTA‐based youth MHA navigation service—developed and implemented a YAC recruitment initiative focused on promoting equity, diversity, inclusion (EDI), accessibility, YE and lived experience/expertise (LE). To adequately summarize the impact of this project, we situate the recruitment approach in the context of FNP's broader Youth Engagement Strategy (YES). The YES was a multi‐year research initiative which aimed at systematically bolstering YE at FNP. It is through the successful implementation of the YES that FNP was able to launch two pilot YAC terms (2021−2023) and build capacity to support this youth‐led project. Finally, to authentically capture the voice of LE, this paper is authored by the youth recruitment team, which includes a youth research coordinator, a youth advocate with lived experience (YAL) (both staff members at FNP), and an active YAC member.

## 2 YAC Recruitment Overview

The goal of the recruitment initiative was to fill 5 of 10 open YAC positions with youth from diverse backgrounds. Initially, we planned to develop and execute our recruitment plan over the span of a couple of months. However, since this was our first time leading a youth‐led recruitment initiative of this magnitude, we opted for a flexible timeline so that the final product was something we were proud of.

Ultimately, the entire recruitment initiative took 7 months to complete (Table 1). Throughout this period, we held weekly meetings, where we discussed progress, next steps, and roadblocks. We connected with the FNP executive director and programme manager on a biweekly basis, mainly providing updates, and when relevant, asking for feedback. By engaging in intimate group discussions with youth peers, and only consulting with management when relevant, we established a youth‐led initiative that was in line with FNP‐Sunnybrook Health Sciences Centre (SHSC) policies and practices.

**Table 1:** Term 3 YAC recruitment timeline.

Timeframe	Phase	Details
September to October 2023	Recruitment planning	The recruitment team developed a timeline for the recruitment process, designed a recruitment poster, selected youth‐friendly organizations/groups to spread the word about the new positions, drafted six application questions and three interview questions and connected with SHSC's communication team to create an application portal.
November to December 2023	Recruitment	The recruitment poster was shared with 49 external organizations/groups via email. It was also shared with current and former FNP clients.
December 2023 to January 2024	Application review	Applications (*n* = 31) were reviewed by the recruitment team. Twelve applicants were selected to be interviewed.
January to February 2024	Interviews	The interviews consisted of one icebreaker and three interview questions. A list of interview topics was shared with applicants ahead of the interviews to help them prepare, and applicants were offered the opportunity to complete either an in‐person or virtual interview.
February to March 2024	Selection and onboarding	Five of the 12 youths were offered a spot on the YAC, filling all available seats. Offer letters were sent via email. Applicants who accepted the position were provided with an onboarding package which included important FNP/SHSC‐related forms, and some youth‐friendly content about the position and upcoming tasks.

*Note:* This table details each phase of the recruitment process, the time it took to complete that phrase and precisely what we did during that stage.

## 3 Insights That Informed the YAC Recruitment Process

At the start of the project, we identified four principles to inform the recruitment process: equity, diversity, and inclusion (EDI); accessibility; YE; and LE (Table 2). These principles were informed by two sources of knowledge: (1) our own LE and positionality as youth who have navigated securing paid and volunteer positions, who have experience navigating their own mental health challenges and/or the care system, as well as experience with prior FNP‐YAC recruitments and (2) extant research on YE. Below, we discuss how these principles guided the recruitment process.

**Table 2:** The four principles that informed the recruitment process.

Topic	Definition	Significance
Equity, diversity and inclusion	Equity, diversity and inclusion (EDI) refers to the removal of systemic barriers and biases to ensure people from different backgrounds are equally valued and respected for their contributions, and equally supported [4].	EDI considerations bring to light whose voices are presently represented in an organization, as well as the types of clients being served. Furthermore, it centres the voices that have been neglected and the types of people and communities not being served.
Accessibility	Accessibility refers to the ease at which a person can reach a literal or figurative destination/goal.	The topic of accessibility emphasizes the logistical considerations needed to include people of diverse backgrounds.
Youth engagement	Youth engagement refers to the organizational practices through which youth participation is made meaningful. An opportunity is meaningful when people/organizations reflect on youth strengths, interests and needs [3].	Reflections on youth engagement highlight the importance of promoting youth autonomy and self‐advocacy in MHA navigation service(s).
Lived experience/expertise	Lived experience refers to the knowledge that is obtained through personal experiences, such as one's experience with mental health challenges, service use and/or recovery [5].	Reflections on lived experience highlight the need to prioritize a broad range of experiences as important and valid sources of knowledge.

*Note:* This table explains the four principles we used to develop our recruitment initiative.

### 3.1 Equity, Diversity, and Inclusion

Throughout the recruitment process, we found ourselves reflecting on the following questions:

1. Who has been historically underrepresented in the MHA field?
2. Who has faced barriers in obtaining work opportunities?
3. Who are we as an organization having difficulties reaching?

These questions led us to consider EDI across the planning, review and selection stages.

To encourage applicants from diverse backgrounds to apply, we made a conscious decision to have minimal eligibility criteria for the position. Beyond the age and geographic restrictions, as per the requirements of the programme, the only restriction we placed was that applicants had to have LE with mental health and/or addiction. This term was not predefined in any of the recruitment material. Rather, we allowed youth to define the term, and youth who self‐identified as having LE with mental health and/or addictions were encouraged to apply. We aimed to further promote diversity through the application process by centering the application questions on applicant's motivation and interest in improving youth access to MHA care. We chose not to include any questions about professional or educational accomplishments, and we opted not to request resumes/CVs. In this manner, emphasis was placed on the youth's perspectives and experiences, rather than accolades that have no significance to the demands of the YAC position.

During the applicant review process, we explicitly looked for applicants' understanding/views on EDI‐related issues. We looked at the value placed on this topic, either from their own LE or simply the recognition of inequality within the system. During the selection process, we continuously reflected on who was currently present and who was missing. We made the intention to prioritize persons from equity‐deserving backgrounds, defined as groups who have been historically denied access to employment, education, and other opportunities. Namely, in the case that there were comparable applicants, we leaned towards applicants from members of equity‐deserving communities. By prioritizing these applicants, we hoped to promote their inclusion in the MHA system and allow their voices to shape FNP practices.

### 3.2 Accessibility

Our goal was to make the recruitment process low barrier for all youth. We tried to accomplish this goal in the application process by implementing a short application form, with only six questions, as well as by allowing applicants the opportunity to either type their answers or to record voice notes of their responses. By limiting the number of questions and allowing for alternative forms of communication, we made the application process more accessible for youth with differing abilities.

During the interview process, we tried to accommodate the diverse accessibility/transportation needs of youth by providing them the choice to complete the interview in person or online through Zoom. To accommodate their busy school/work schedules, we offered a wide range of interview time slots, including time slots before 9 AM and after 5 PM. If the allotted times did not work, we offered applicants the opportunity to request alternative times. To further set applicants up for success, we emailed them a list of interview topics 1 day before the interview. We anticipated that this approach would help mitigate the anxiety associated with interviews, especially for youth who may not have prior interview experience.

We sought to improve accessibility during the interview stage by communicating interview questions both verbally and in writing (via Zoom chat box). We also offered online applicants the ability to turn on closed captions. These small logistical tweaks were incorporated to promote the success of people with diverse abilities.

### 3.3 Youth Engagement

The initial development of the YAC was informed by FNP's YES [5]. This strategy aimed to improve YE efforts and ensure the youth voice can shape FNP's service. The guiding principles of the strategy include:

1. Deepening connections with youth across the GTA,
2. Youth guiding FNP, and
3. Optimally serving youth.

Drawing on learnings from this strategy, we deemed it necessary to reach out to youth, rather than waiting for them to connect with us. We did this by not only sharing the opportunity with our own personal and professional networks and current and former FNP clients but also with people who work with youth in youth‐friendly spaces. We determined the youth‐friendliness of an organization based on personal encounters with the organizations, feedback from previous clients and how the organizations displayed their value of diversity and LE through their social media accounts and/or websites. We asked these organizations to spread the word through word of mouth, physical posters, and if they had social media presence, through social media posts.

At the interview stage, we sought to increase YE by promoting youth‐friendly approaches. For instance, we intentionally designed the interview panel to include only youth and to ensure each interview included no more than three questions. Additionally, we started each interview with an icebreaker question, allowing both the interview panel and the applicants to ease into the conversation. The goal of the interview was to get to know applicants and learn about their perspectives and motivations as they relate to the YAC role. By promoting youth‐friendliness, we were able to mitigate power dynamics, allowing applicants to be more comfortable, and thus able to show up as their best selves.

The understanding of YE put forth by the YES encouraged us to reflect on ways of promoting growth and development. This consideration was reflected in the application form and interview process, where youth were asked to share how they would contribute to the position, as well as what they aimed to get from this position. This information was used not only to help decide whether this position was a good fit but also to inform future YAC‐related processes and ensure mutual benefit for both FNP and YAC members.

### 3.4 Lived Experience

Within this recruitment initiative, LE/expertise was understood as a valid and valued knowledge base, which we deemed of greater value to the YAC than any form of formal education or professional experience. This idea was reflected across our recruitment process through the inclusion of self‐identified LE as the main eligibility criteria, and in the applicant selection process, where applicants who focused on speaking from a LE perspective were prioritized.

Having completed the recruitment process, we believe that our decision to omit a definition of LE positively impacted our recruitment process. Specifically, we believe this decision allowed for youth with a range of MHA‐related experiences and encounters with the MHA system to apply. Of particular interest was a group of youth who reported lived/living experiences engaging in the MHA care system through a caregiving role. We found these perspectives to be very interesting, as we had not initially considered them when reflecting on the types of youth perspectives that would be beneficial for our YAC. These types of responses validated our decision to allow youth to define LE, as it seemed to encourage application from people we would have missed out on if we had provided a rigid definition.

An unforeseen challenge that appeared in our attempt to promote LE occurred at the initial planning stage when we were developing application and interview questions. We spent a lot of time trying to figure out how to showcase our value for LE without pressurizing youth to disclose personal information about their life experiences. Through on‐going discussions about the type of information that was necessary for our decision‐making process, we ended up finding a comfortable middle ground. Specifically, we ended up developing a set of application and interview questions that focused on applicants' opinions, interests, and motivations, allowing them to discuss only those of their LE that they deemed relevant to the role—if they felt comfortable doing so.

## 4 Recruitment Outcomes

When reflecting on the 7‐month project, we are satisfied with the results of our careful planning and intentionality regarding EDI, accessibility, YE, and LE. We were able to successfully recruit five new YAC members. The new YAC members included youth who identified as recent immigrants, racialized, and as a part of the 2SLGBTQIA+ community. In terms of location, youth were from various neighbourhoods across the GTA. The youth offered unique perspectives when it came to what MHA looked like or meant to them. Through the application and interview process, they highlighted important differences in what MHA means in a Western context compared to an Eastern context. They also discussed how MHA challenges influenced them personally, as well as how they shaped their family dynamics.

When reflecting on our recruitment processes, we recognized multiple opportunities to enhance the accessibility and equitable experiences of interviews in the future. For example, letting participants know, up front, the importance of being in a private space for the interview will help ensure comfort and ability to share their experience. Furthermore, letting them know that interviews can easily be rescheduled can prevent stress as a result of unforeseen circumstances. During interviews, closed captions can automatically be enabled to help promote universal accessibility. To adhere to recruitment timeframes, all interviews were completed within 1 week. Although many times were offered outside regular business hours, condensing interviews into 1 week limited the interview slots that could be offered to candidates and may have caused them to forego other commitments to participate in the interview.

Another future opportunity exists with respect to distribution of roles and responsibilities. The youth recruitment team handled the main responsibilities of this project, and mainly connected with members of the broader FNP team to share updates. While this allowed the project to be youth‐led, there were moments where additional support from the leadership team could have been helpful. Identifying and communicating support needs will be important in future recruitment rounds. In the future, we believe it is important to clearly establish roles ahead of time. Lastly, we recognize that this project was spearheaded by three youths. Although the three of us come from diverse backgrounds, we understand we do not represent all youth. For the next YAC recruitment, we believe it is important to introduce new youth to the recruitment team. This will allow for more diverse perspectives to be integrated into the decision‐making process.

## 5 Conclusion

This paper provides an overview of an approach to YAC recruitment that emphasizes EDI, accessibility, YE, and LE. We highlighted how a youth‐led recruitment initiative allowed for the recruitment of diverse youth across the GTA. The decisions and reflections noted in this paper were specific to the FNP context and are by no means flawless. As such, these should not be considered a prescriptive process to be implemented by other organizations. Rather, these reflections may serve as a discussion point and source of inspiration for MHA and other navigation services. Particularly, these organizations should reflect on the utility of EDI, accessibility, LE, and YE‐related considerations in the development of YACs and for the purposes of better serving youth across the MHA system.

## Author Contributions

**Deewa Anwarzi:** conceptualization, investigation, writing – review and editing, writing – original draft. **Nhi Man (Jennifer) Tran:** conceptualization, investigation, writing – review and editing, writing – original draft. **Thalia Phi:** conceptualization, investigation, writing – original draft, writing – review and editing. **Roula Markoulakis:** funding acquisition, supervision, resources, writing – review and editing.

## Conflicts of Interest

The authors declare no conflicts of interest.

## Data Availability

The authors have nothing to report.
